# Circuits of cancer drivers revealed by convergent misregulation of transcription factor targets across tumor types

**DOI:** 10.1186/s13073-015-0260-1

**Published:** 2016-01-20

**Authors:** Abel Gonzalez-Perez

**Affiliations:** Research Program on Biomedical Informatics, IMIM Hospital del Mar Medical Research Institute and Universitat Pompeu Fabra, Doctor Aiguader 88, 08003 Barcelona, Spain

## Abstract

**Background:**

Large tumor genome sequencing projects have now uncovered a few hundred genes involved in the onset of tumorigenesis, or drivers, in some two dozen malignancies. One of the main challenges emerging from this catalog of drivers is how to make sense of their heterogeneity in most cancer types. This is key not only to understand how carcinogenesis appears and develops in these malignancies to be able to early diagnose them, but also to open up the possibility to employ therapeutic strategies targeting a driver protein to counteract the alteration of another connected driver.

**Methods:**

Here, I focus on driver transcription factors and their connection to tumorigensis in several tumor types through the alteration of the expression of their targets. First, I explore their involvement in tumorigenesis as mutational drivers in 28 different tumor types. Then, I collect a list of downstream targets of the all driver transcription factors (TFs), and identify which of them exhibit a differential expression upon alterations of driver transcription factors.

**Results:**

I identify the subset of targets of each TF most likely mediating the tumorigenic effect of their driver alterations in each tumor type, and explore their overlap. Furthermore, I am able to identify other driver genes that cause tumorigenesis through the alteration of very similar sets of targets.

**Conclusions:**

I thus uncover these circuits of connected drivers which cause tumorigenesis through the perturbation of overlapping cellular pathways in a pan-cancer manner across 15 malignancies. The systematic detection of these circuits may be key to propose novel therapeutic strategies indirectly targeting driver alterations in tumors.

**Electronic supplementary material:**

The online version of this article (doi:10.1186/s13073-015-0260-1) contains supplementary material, which is available to authorized users.

## Background

Cancer develops as a consequence of the accumulation of driver somatic alterations in genes which, in turn, modify critical cellular processes often referred to as the hallmarks of cancer [1, 2]. The catalog of driver genes involved in the development of several malignancies has grown in recent years, as a result of whole-exome and whole-genome analyses of cohorts of tumors, mainly within the framework of large international consortia [3, 4]. This has opened up the possibility to carry out systematic studies to uncover the repertoire of functionally related groups of driver genes. Exploiting the aforementioned catalogs of driver genes, for example, we recently revealed the mutational landscape of chromatin regulatory factors (CRFs) in cancer [5]. Driver genes have also been grouped by their proximity in a network of functional interactions or their membership to the same or cross-talking pathways [6–9].

There are two main reasons why the catalogs of cancer drivers produced by the aforementioned projects and others need to be broken down into related sets of genes for deeper analysis. First, while the most frequent drivers in these lists have been long known and studied in their involvement in tumorigenesis, many novel mid- and low-frequent drivers have emerged whose roles in cancer need to be systematically clarified. One step in this direction is thus to understand exactly which downstream genes and cellular processes become affected in the outcome of driver alterations. Second, uncovering the catalog of driver genes has revealed that at the level of genomic alterations, tumorigenesis possesses a very heterogeneous nature, with driver alterations in genes in the same pathway or in cross-talking pathways resulting in the same phenotype [2, 6–8]. This is the case, for instance, of loss-of-function mutations of *TP53*, amplifications of *MDM2* and *MDM4*, and deletions of *CDKN2A*, all leading to evading apoptosis and uncontrolled proliferation in malignancies such as glioblastoma. Such cliques of drivers, suspect of producing the same set of downstream changes have been identified using algorithms that take advantage of their trend to be altered in a mutually exclusive manner across tumor samples [6–8]. Nevertheless, to date, the actual misregulation of genes or processes downstream these cliques has not been systematically proven or exploited with the purpose of identifying them. The heterogeneity of driver alterations in a tumor type presents a major challenge to the efforts to develop a comprehensive toolbox of targeted therapies to extend personalized cancer medicine driven by genomics information. In a recent study we showed that only 6 % of the tumors in a cohort of more than 4,000 could be treated employing currently approved targeted therapies [10].

In this work I have chosen transcription factors (TFs) as a case study to try to make sense of the heterogeneity of drivers in tumor types through their downstream alteration for two main reasons. First, several human TFs controlling the expression of sets of target genes involved in the hallmarks of cancer, as *TP53* or *MYC*, are frequently involved in tumorigenesis upon somatic alterations. Second, because TFs directly regulate the expression of groups of genes (targets), one may intuitively think that the tumorigenic effect of their driver alterations could be measured by computing the level of misregulation of these targets. Here, I start by uncovering and describing 64 TFs involved in tumorigenesis across 28 malignancies. Then, to investigate how their somatic alterations result in cancerogenesis across 15 tumor types, I explore the misregulated targets of 42 of them for which I was able to gather data. Lists of significant targets for different TFs in the 15 tumor types under study are thus provided as an outcome of this work. Moreover, employing the sets of significant targets of driver TFs, I search for other connected drivers whose alterations result in the misregulation of significantly overlapping sets of genes. These driver circuits involving a TF and another connected driver (or partner) are also provided as an outcome of the study.

## Methods

### Detection and analysis of the repertoire of driver transcription factors

I obtained the list of human TFs employed in this study from a catalog compiled by Vaquerizas *et al*. [11]. I retrieved all TFs rated as ‘a’, ‘b’, or ‘other’, which the authors refer to as a high-confidence dataset. This list comprised 1,391 TFs. From this paper I also obtained the information on all InterPro [12] domains of these 1,391 TFs, including the identifier and name of the domain, as well as its boundaries and its source database. I inherited the list of mutational driver genes across 28 malignancies from our recent study [10], which identified them on the basis of three methods that exploit complementary signals of positive selection of the pattern of mutations in the genes in the tumorigenic process [10, 13]. This catalog of mutational drivers included 459 genes. It also contained the most likely mode of action of each driver (loss-of-function (LoF) and activating (Act)), predicted by a random forest classifier trained on known tumor suppressors and oncogenes [14]. Unclassified driver TFs were considered for all relevant purposes as Act drivers. All drivers nominated by the aforementioned study are known to be expressed either in the tumors of the cohort where they act as drivers (in the case of TCGA cohorts), in a cohort of tumors of the same cancer type or in a cohort of tumors from the same organ [10, 13].

I retrieved all mutations detected in driver TFs across almost 7,000 tumors sequenced in 48 cohorts representing the 28 aforementioned tumor types from the Integrative Oncogenomics (IntOGen) platform [15] (www.intogen.org). In the datasets downloaded from IntOGen, mutations had been already mapped to the amino acid positions of the affected proteins. I then computed the enrichment/depletion of mutations in each driver TF in each cohort of tumors as the Fisher’s test *P* value.

I downloaded all germline variants detected across 65,000 exomes of different cohorts of donors from the database collected by the Exome Aggregation Consortium (ExAC), Cambridge, MA (http://exac.broadinstitute.org, downloaded in November 2014) [16]. Again, the data provided by the ExAC already comprised the amino acid coordinates of variants. I filtered out all variants with allele frequency below 10^–4^. For each domain of each TF, I counted the number of variants and somatic mutations observed within and outside the domain, and computed the relative enrichment of each domain for somatic mutations using Fisher’s test.

### Detection of misregulated targets of TFs

I collected a list of targets of TFs gathering information from several databases. From HTRIdb [17], pazar [18], and TRANSFAC v. 7.4 via MSigDB [19, 20], I manually downloaded annotated targets of 283, 190, and 283 TFs, respectively. From the Additional Data of the paper by Gerstein *et al.* [21], I obtained targets detected by ENCODE, including both proximal and distal sites.

To detect all putative driver alterations (mutations, amplifications, and deletions) across 15 TCGA cohorts of tumors (Additional file 5), I first downloaded CNA (continuous values per genomic segment) and expression data (processed RNAseq in the form of RPKMs) from synapse (syn300013). Also from synapse, I retrieved the mutations detected in 34 pancreatic carcinoma samples, not included within the datasets of somatic mutations obtained from IntOGen. Then, I declared the alteration of a driver TF in a tumor sample a driver alteration according to the following rules. (First, because drivers – and by extension driver TFs are defined by tumor type – the process described below was carried out for the driver TFs of each tumor type independently.) Both truncating – stop gained or lost, frame-shift, splice donor or splice acceptor – and missense mutations in LoF driver TFs were considered LoF driver mutations, while only missense mutations in Act driver TFs were considered Act driver mutations. To detect driver amplifications and deletions, I first identified LoF driver TFs whose deletions (CNA values below –0.75) caused a downregulation of their mRNAs and Act drivers whose amplifications (CNA values above 1) caused an upregulation of their mRNAs. Upregulation and downregulation were determined as significant (*P* value <0.05) Mann–Whitney comparisons of the expression of each driver TF in samples with deletions –or amplifications – to samples with a number of copies close to normal (between –0.75 and 1). Then, I declared every deletion involving an LoF driver TF a driver deletion, and each amplification involving an Act driver TF a driver amplification. These were merged with LoF and Act driver mutations, respectively, to produce the final matrices of samples with driver alterations of each driver TF in each tumor type.

The expression of each target in the samples bearing driver alterations of the TF and unaltered samples were then compared using a Mann–Whitney test. From this comparison I excluded the samples bearing either amplifications or deletions of the target, according to the criteria mentioned above. I considered significant targets those with *P* value of the comparison below 0.05 and fold-change (log2) above 1 or below –1. The aim of this differential expression test is – regardless of the high overlap observed between the targets of TFs and the top-ranking misregulated genes in response to their knock-down – to identify the subset of targets of each TF which are actually misregulated upon alterations of the TF within the context of the tumor type under analysis. In this case, I compared the number of differentially expressed genes among the targets of a TF with differentially expressed genes detected within groups of randomly selected genes of the same size as the annotated targets. The results show a varying landscape, with some TF-tumor type combinations for which misregulated genes are mostly confined to the annotated targets, and others where differentially expressed genes are distributed both within and outside the targets. These are probably the result of incomplete annotation of TF targets, or of drastic changes in the transcriptional regulatory program in tumorigenesis.

To determine the coincidence of the sets of significant targets of each driver TFs in a pair of tumor types I computed two metrics, the Jaccard’s index and the corrected (Benjamini–Hochberg) Fisher’s *P* value (q-value) of their overlap. The universe used to compute the latter was the whole list of potential targets of the TF collected from the four original sources.

To compare the effect of truncating and missense mutations of TP53 on the expression of its targets, I pooled the absolute fold-change values of all targets in the comparison between samples bearing truncating mutations – or missense-mutations – of the TF and non-altered samples. I then compared both distribution of absolute fold-change with a Mann–Whitney test.

### Detecting driver circuits

I downloaded the PathwayCommons2 Functional Interactions network [22] (version 6 downloaded in November 2014). I retained interactions between genes of types ‘in-complex-with’, ‘interacts-with’, ‘neighbor-of’, ‘controls-phosphorylation-of’, and ‘controls-state-change-of’ and discarded the rest. For every TF-tumor type combination I searched all connected non-TF drivers (partners) with no more than half of the samples with driver mutations overlapping between TF and partner. For each TF-partner pair I assessed whether the alterations of the partner cause tumorigenesis through the same pathway as alterations in the connected TF. To that end, I first repeated the search for significant targets detailed in the previous section comparing this time the expression of targets of the TF in samples bearing driver alterations of the partner and samples where neither the partner nor the TF bore driver alterations. This way I ensured that targets deemed significant in this search are not the result of alterations of the TF. As a proxy to the strength of the link between partner and TF in their causing tumorigenesis through alteration of the same cellular pathway, I computed the corrected (Benjamini–Hochberg) Fisher’s q-value of the overlap between the significant targets of alterations of the TF and the significant targets of the alteration of the partner.

To carry out the sample level enrichment analysis (SLEA) [23] of upregulated and downregulated targets of sample TF-partner circuits, I first transformed the expression of targets to mean-centered values. Then, I employed the SLEA built-in capability of Gitools [24]. All heatmaps presented across figures were also constructed, configured, and exported using Gitools.

### Assessing mutual exclusivity of alterations of the members of driver circuits

I employed two recently published methods [25, 26] to assess the mutual exclusivity of alterations of groups of genes. I downloaded both mutex and Comet from the sites provided by the authors (http://code.google.com/p/mutex and http://compbio-research.cs.brown.edu/comet, respectively) and constructed and ran them as documented. In both cases I ran them on the set of driver alterations detected in the tumor datasets employed to detect the circuits explored in this work. In the case of Comet, I ran the run_exhaustive.py script.

For both tools I limited the search for mutually exclusive genes to size two and filtered the altered genes to take into account for mutual exclusivity by the list of TFs and partners integrating the circuits assessed in each tumor type. I then categorized the mutual exclusivity of all potential driver circuits probed in this work (with at least one significantly misregulated downstream target gene) according to the results of both methods.

## Results

Additional file 1: Figure S1 presents an overview of all the analyses carried out in this study.

### The repertoire of driver transcription factors across 28 tumor types

**Fig. 1:**
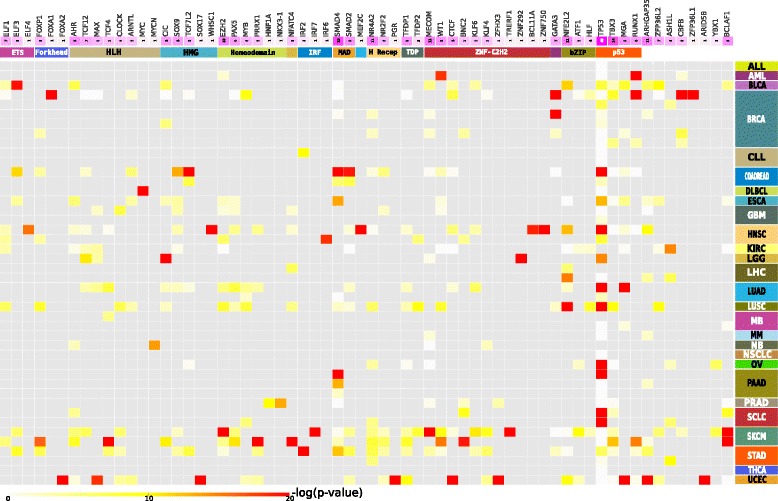
Enrichment for mutations of driver TFs across the 48 cohorts of tumors. The color scale of the cells follows the -log(*P* value) of Fisher’s test (see color legend at the bottom). Column color annotations from top to bottom represent the number of cohorts where the TF is identified as driver (the darker the purple, the more cohorts) and the family of the TF. The abbreviations used, and full names of families of each TF appear in Additional file 2: Table S1. Row color annotations represent the cohorts of tumors where the driver TFs were detected, with different colors symbolizing cohorts of tumor of different tumor types, the acronyms of which are detailed in the list of abbreviations. The source of mutational data of each cohort appear in Rubio-Perez *et al*. (20015) [10]

We had previously carried out an exhaustive analysis of whole-exome mutations of 48 cohorts of tumors obtained from 28 different malignancies (approximately 7,000 tumors in total) employing three computational methods that exploit complementary signals of positive selection and identified 459 mutational driver genes [10]. I started with this list of cancer driver genes obtained from the IntOGen platform. TFs are overrepresented among them (*P* value = 3.9 × 10^–8^), with 64 proteins (Fig. 1 and Additional file 1: Figure S2) identified from a catalog of 1,391 human TFs [11]. I also retrieved the most likely mode of action of each driver TF upon alterations in tumorigenesis (either activating, LoF, or undetermined) across approximately 4,000 TCGA samples, determined by a random forest classifier trained on the pattern of somatic alterations of known drivers [14]. For example, an activating driver TF (such as MYC) is expected to be more active than normal when affected by gain-of-function mutations or amplifications, and the opposite for a LoF TF driver, such as TP53 when affected by LoF mutations or deletions. Note that this classification has no relation whatsoever with the effect of the TF on the expression its targets, a set of which may result up- or downregulated irrespective of the type of alterations suffered by the driver TF. According to this list, there are 22 Act and 36 LoF driver TFs, as well as six driver TFs with undetermined mode of action. Members of 15 TF families [11] participate in tumorigenesis in the 28 tumor types analyzed. Twelve of them are represented by more than one driver protein (Fig. 1 and Additional file 2: Table S1). The largest family of TFs (Zinc fingers C2H2) also contributes the largest share of drivers to tumorigensis in the 28 cancer types.

The 64 driver TFs possess varying degrees of implication in tumorigenesis in different tumor types, as illustrated in Fig. 1 and Additional file 1: Figure S2. For instance, while TP53 is significantly enriched for mutations in ovarian, colorectal, head and neck, or lung cancers, it is mutated below expectations – set by other driver TFs – in thyhroid and prostate carcinomas, as well as in melanomas and medulloblastomas. The activity of some TFs as mutational drivers is very specific of one or few tumor types. This is the case of *GATA3* in breast tumors, *ZNF292* in low grade gliomas, *ARID5B* in uterine carcinomas, and *MYC* in diffuse B-cell lymphomas. Well-known driver TFs such as *TP53*, *SMAD4*, and *NFE2L2* acting in 40, 15, and 11 cohorts, respectively, share the top-ranking positions with others just recently identified, like *TBX3* (14 cohorts), which promotes cell migration and invasion [27], *MECOM* (11 cohorts), with inhibitory effects on apoptosis [28], or *ARHGAP35* (11 cohorts), a GTPase-activating protein that promotes invasion and metastasis [29].

In an effort to identify the domains of each TF that are most relevant to tumorigenesis upon somatic mutations (Additional file 1: Figure S1B), I computed the relative enrichment of each domain (defined by InterPro [12]) for somatic mutations (SNVs and short indels) across the approximately 7,000 tumors in the cohort versus common variants observed in the exomes of 65,000 individuals (see Methods). In the case of somatic mutations detected across tumor samples, only those with consequence-types deemed to affect the protein sequence (that is, missense, stop-gain, stop-loss, splice-donor, splice-acceptor, frameshift) were included. In the case of the variants detected across non-cancer genomes of individuals, I included SNVs and short indels with all consequence types, but only those with allele frequency above 10^–4^, which ensures that they are likely to be common polymorphisms in the human population.

**Fig. 2:**
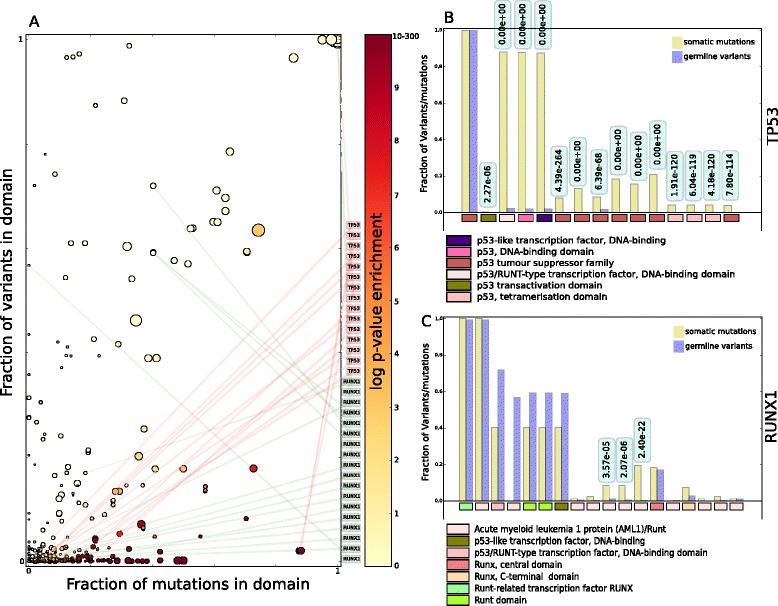
Relative enrichment for somatic mutations of driver TFs domains across approximately 7,000 tumors. **a** InterPro domains of all driver TFs represented in a space of fraction of variants (with respect to the total in the protein) versus fraction of mutations. The diameter of circles is proportional to the length of the domains (number of amino acids) they represent, and their colors follow the scale for -log(*P* value) represented at the right of the panel. TP53 and RUNX1 domains are highlighted. (To facilitate visualization of enriched domains the color scale of log *P* values of enrichment is truncated at 10.) All data included in this figure are provided in Additional file 3: Table S2. **b**, **c **Barplot representation of the fraction of mutations (amber) and germline variants (blue) that appear within each domain of TP53 and RUNX1. Each pair of bars correspond to a domain, represented below the graph following the color code presented at the bottom of both graphs. Relative enrichment *P* values (corresponding to the position of the circles in panel (**a**) appear at the top of each domain significantly enriched for somatic mutations

This analysis therefore aimed to rank higher domains with the greatest differences between the accumulation of somatic mutations in tumors that signals them as subjects of positive selection and the number of common germline variants that entail their baseline tolerance to variation. I thus computed the fraction of mutations occurring in every domain of a TF out of the total number of mutations observed in the gene across tumors and, similarly their share of common germline variants. Each circle in Fig. 2a corresponds to a TF domain represented in a plane defined by these two quantities, whereas its color represents the significance of its relative enrichment for somatic mutations. All domains of *TP53* (annotated at the right of panel A) accumulate a relatively low fraction of the variants observed in the gene, except the p53 tumor suppressor family, which encompasses most of the protein sequence. The fraction of mutations occurring at each domain is nevertheless significantly higher (Fig. 2b). Whereas somatic mutations impairing the activity of *TP53* tend to accumulate in the DNA-binding domain and the tetramerization domain of the TF, tolerated germline variants concentrate in the N-terminal and C-terminal portions of the protein, outside its most important structural and functional features. In the case of *RUNX1* (Fig. 2c) somatic mutations tend to accumulate in the short stretches of sequence that define the AML1/Runt domain. Although many of the domains that appear at the top of the list of relative enrichment for somatic mutations correspond to the portions of the TF that directly bind the DNA (p53, DNA-binding domain, winged helix-turn-helix, zinc finger, homeodomain, and so on), other types of TFs domains are also tumorigenic. The latter include the aforementioned tetramerization domain of *TP53* and the ligand binding domain of *NR4A2*, a nuclear hormone receptor (Additional file 3: Table S2).

### Pan-cancer misregulation of genes under the control of driver TFs

The rationale of this study – the hypothesis that driver alterations of TFs result in misregulation of their targets – assumes that driver TFs are expressed and carry out their functions in the tumor types where they are identified as drivers and where I probe this misregulation. The list of drivers employed in this study in each tumor type (and by extension the driver TFs) are known to be expressed in the tumors of the cohort where they act as drivers, because part of the process of detection of mutational drivers excludes non-expressed genes. As for their activity, driver genes are detected as such because they exhibit signals of positive selection (see references [10] and [13]) in the tumor types where they are nominated as drivers, which implies that either their over-activation (in the case of oncogenes), or their inactivation (for tumor suppressors) exists and is key to the development of tumorigenesis. (Note that in the latter case, the inactivation in tumors bearing LoF alterations presupposes that they are active in the other tumors of the cohort).

To investigate the changes in expression of genes resulting from the alteration of driver TFs (Additional file 1: Figure S1C), I first collected lists of genes under the regulation (targets) of TFs from four sources (Additional file 1: Table S3). These four online resources contain curated TF targets retrieved from the literature (HTRIdb [17], pazar [18], and TRANSFAC v. 7.4 via MSigDB [19, 20]), and the list of genes with either proximal or distal TF binding sites detected through ChIP-seq analysis by the ENCODE consortium [21]. Forty-two driver TFs were assigned targets (ranging from 1 to 5,963 with median 232; Additional file 1: Table S4) retrieved from one or more of these databases. In every tumor sample within 15 cohorts analyzed by TCGA (Additional file 1: Table S5), I then determined the alteration status of each driver TF. In this case, because the copy number status of each gene is available besides the list of somatic SNVs and short indels (hereinafter, mutations), I employed both types of alterations to define the samples where each driver TF is altered. Missense mutations and amplifications were considered driver alterations of activating driver TFs. On the other hand, driver alterations of LoF driver TFs are truncating or missense mutations or deletions (see Methods). Driver TFs with undetermined mode of action were applied the same rule as activating driver TFs. Finally, I compared the expression level of the target of each driver TF in the tumor samples bearing driver alterations with that of the samples where it was unaltered. A target was considered significantly misregulated (misregulated targets) upon driver alterations in a TF if the Mann–Whitney *P* value of the comparison was smaller than 0.05 and the log2 of the fold-change was either smaller than –1 or greater than 1. Note that because the targets obtained from the four aforementioned sources have been detected irrespective of the tissue type and normally in healthy tissues, the differential expression analysis acts as a filter to detect those targets most likely misregulated upon alterations of driver TFs.

**Fig. 3:**
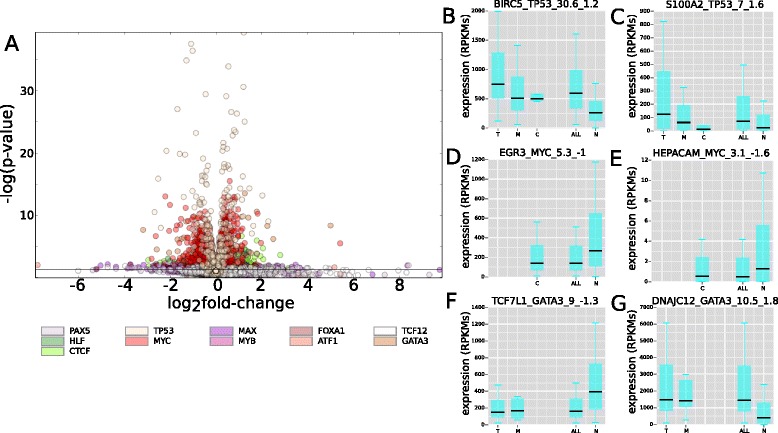
Significant targets of driver TFs in the breast cancer cohort. **a** Volcano plot with the results of the differential expression analysis. Each circle represents a target gene placed in Mann–Whitney -log(*P* value) versus log2(fold-change) coordinates. Targets of each TF are represented following the color code represented below the graph. Volcano plots for other tumor types appear in Additional file 1: Figure S3. All data presented in these figures are provided in Additional file 4: Table S6. **b**-**g** Distribution of the expression of six illustrated targets of TP53 (**b**, **c**), MYC (**d**, **e**), and GATA3 (**f**, **g**). Each boxplot in the graphs correspond to the expression of the target in samples bearing one type of driver alteration – truncating (T) and missense mutations (M), and deletions (C) in the case of TP53 and GATA3, and amplifications (C) in the case of MYC – any driver alteration (ALL), or no driver alteration (N). Boxplots are only represented for sets of at least five samples. The name of each graph is formed by a concatenation of the name of the target gene, the name of its TF, the Mann–Whitney -log(*P* value) and the fold-change of its differential expression analysis

The volcano plot in Fig. 3a illustrates the distribution of targets in a *P* value to fold-change plane resulting from the differential expression analysis of all driver TFs in the breast cancer cohort (BRCA), with the lines delimiting significance highlighted. Similar volcano plots and results of the analysis in other tumor types appear in Additional file 1: Figure S3 and Additional file 4: Table S6. The distribution of the expression of six misregulated BRCA targets are shown in panels (b) to (g) of Fig. 3. BIRC5 or survivin (Fig. 3b), a target of *TP53* is an anti-apoptotic molecule that has been previously linked to the development and progression of breast cancer and the emergence of drug resistance, as well as with tumorigenesis in other cancer types [30–32]. *S100A2*, which also appears upregulated in samples bearing driver alterations of *TP53* (Fig. 3c) has been related to tumor promoting processes, such as the epithelial-mesenchymal transition. Initially, though, to act as a tumor suppressor, it probably plays a dual role in cancerogenesis [33–35]. In both cases, truncating mutations of *TP53* appear to cause larger upregulation of the targets, supporting the notion that they possess a higher driver potential, with some missense mutations actually acting as passengers. Nevertheless, the pooled analysis of the misregulation of all targets of *TP53* across all tumor types reveals that although in most cases truncating mutation of the TF do affect more the expression of its targets than missense mutations, the differences are clearly significant only in breast tumors, glioblastomas, and low grade gliomas (Additional file 1: Figure S4).

*EGR3* and *HEPACAM* are significantly downregulated in samples with *MYC* amplifications (Fig. 3d and e). The former encodes an early growth response TF related among others to lymphocyte development and endothelial cell growth and migration whose downregulation could play a role in the proliferation, metastasis, and progression of cancer cells [36, 37], whereas the latter is a likely tumor suppressor gene which codes for a protein involved in cell motility [38]. Finally, panels F and G illustrate the misregulation of *TCF7L1* and *DNAJC12*, two significant targets of *GATA3* linked to tumorigenesis. *TCF7L1* encodes a TF in the WNT pathway which regulates the expression of cell cycle related genes and is a central regulator of tumor growth and initiation [39–41]; *DNAJC12* is a chaperone upregulated in several cancer types.

**Fig. 4:**
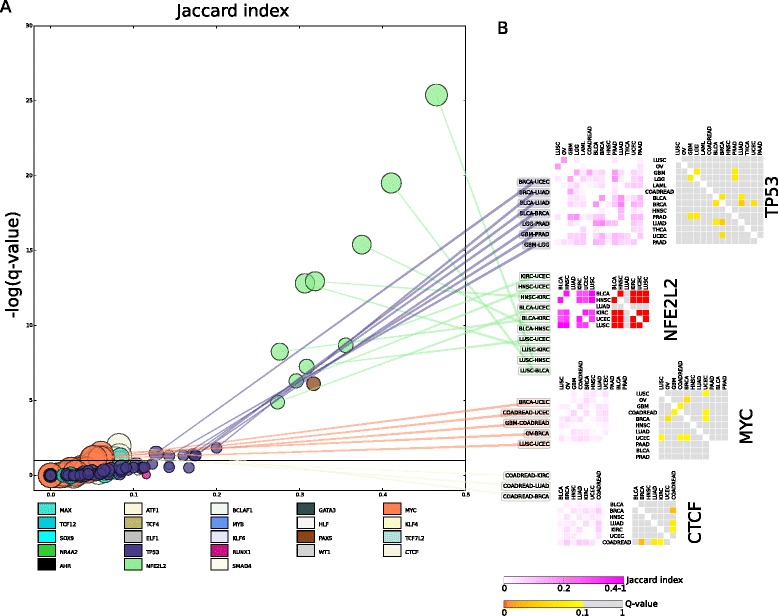
Overlap between significant targets of each driver TF across tumor types. **a** Pairs of tumor types represented in a plane of the Jaccard’s index of the overlap of significant targets versus its significance computed as the Fisher’s -log(q-value). Overlaps between sets of targets of each TF are represented following the color code shown at the bottom of the graph. The diameter of the circles is proportional to the size of the overlapping list of targets. Selected pairs of tumor types with significant overlap of targets of different driver TFs appear at the right of the graph. **b** Overlap of significant targets of selected TFs between pairs of tumor types where they act as drivers. Heatmaps at the left contain the Jaccard’s index of the overlap between significant targets detected in each tumor type, while heatmaps at the right represent the Fisher’s -log(q-value) of each comparison. Color scales for both magnitudes appear at the bottom of the panel. All data presented in these figures are provided in Additional file 5: Table S7

The sets of significant targets of most driver TFs differ widely between tumor types (Fig. 4a and Additional file 5: Table S7), with the notable exception of *NFE2L2* and, to some extent, *TP53*, *MYC* (Fig. 4b), *PAX5*, and *RUNX1*. Nineteen genes are significantly over-expressed in samples of five tumor types driven by somatic alterations of *NFE2L2*. These include a set of enzymes involved in the antioxidant response promoted by this TF, such as *NOQ1*, *AKR1B10*, *AKR1C1*, *AKR1C3*, and *ALDH3A1*, but also the glucose-6-phosphate dehydrogenase, whose upregulation sustains the elevated production of NADPH and biosynthesis required by tumor proliferation [42]. *NFE2L2* thus probably causes the shift of the cellular metabolic program to promote tumorigenesis in cancer types driven by its alterations. This implies that tumors driven by alterations of *NFE2L2*, regardless of their tissue of origin are much more similar to each other than tumors driven by alterations of other driver TFs.

The coincidence of significant targets of TP53 is also significant across several malignancies. Significant overlap occurs between the sets of targets in tumors of the central nervous system (GBM and LGG) and prostate adenocarcinomas. These include the over-expression of *COL11A1*, typical of mesenchyme-derived tumors with high metastatic potential [43], and the under-expression of *PHLDA3*, a protein with a PH domain which inhibits the activation of Akt [44]. A second group of tumor types with significant overlapping targets include bladder, breast, lung, and uterus carcinomas (Fig. 4b).

### Circuits of drivers connected to TFs

Finally, I exploited the sets of significant targets of driver TFs to find other drivers in the same tumor type causing similar downstream alterations, under the hypothesis that somatic alterations of different drivers in the same or cross-talking pathways may converge in the downstream alteration of the expression of similar groups of genes (Additional file 1: Figure S1D). To this end, I first searched for all drivers connected to each driver TF (partner drivers) in each tumor type in a network of functional interactions [22] with no more than 50 % of their altered samples overlapping with those of the TF. I then repeated the process of identifying significantly misregulated genes – within the set of targets of the TF – between the group of tumor samples bearing driver alterations of the partner and the tumors where neither the partner nor the driver TF were altered. Next, I computed the significance of the overlap between the set of upregulated genes upon driver alteration of the TF and the set of upregulated genes upon driver alteration of the partner, and the same for the sets of downregulated genes. I used the two corrected *P* values of the overlaps between upregulated and downregulated sets of genes as a proxy of the strength of the tumorigenic link between the TF and the partner in tumorigenesis. I call these connections TF-partner ‘driver circuits’ – driver circuits in this paper, for simplicity – and understand them as common pathways towards tumorigenesis, with driver alterations of their two members resulting in convergent misregulation of the same cellular processes.

**Fig. 5:**
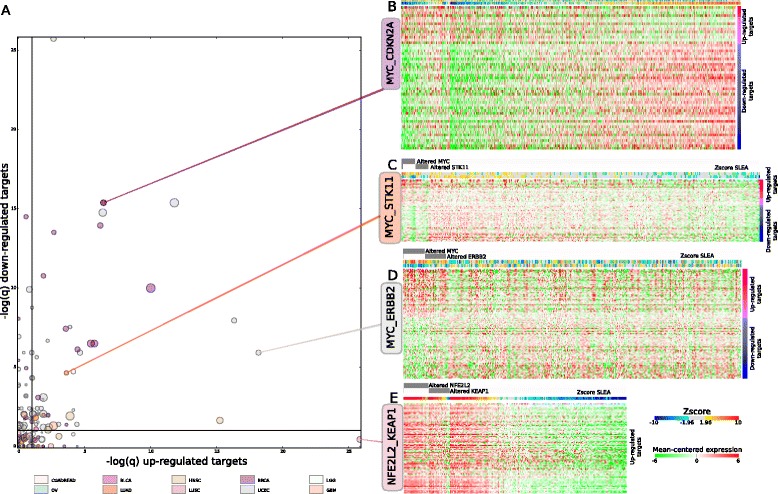
Circuits of drivers involving TFs. **a** Driver partner-TF circuits (circles) displayed according to the significance of the overlap of their downregulated targets versus the significance of the overlap of their upregulated targets. The diameter of each circle is proportional to the average size of the overlapping upregulated targets and downregulated targets. The colors of circuits follow the code of their respective TF shown at the bottom of the graph. All data presented in this graph are provided in Additional file 6: Table S8. **b**-**e** Examples of the misregulation of targets caused by partner-TF circuits in uterus (**b**), breast (**c**), lung (**d**), and head and neck (**e**) carcinomas. In each heatmap, target genes are represented as rows and tumor samples are represented as columns. Cells represent the mean-centered values of expression of each target in each tumor sample (color scale below panel **a**). Upregulated targets appear at the top of the heatmap (purple block), while downregulated targets appear at the bottom (blue block). The uppermost layer of annotations above the columns of the heatmaps signal samples bearing driver mutations of the TF (top row) and the partner (bottom row). The second layer of annotations present the collective degree of alteration of upregulated (top row) and downregulated (bottom row) targets in each tumor samples, determined as the Zscore of the Sample Level Enrichment Analysis (SLEA) of both sets of targets (color scale below panel **a**). Samples in the heatmap are sorted following the mutual exclusivity pattern of driver alterations in the TF and the partner

*TP53* and *MYC* possess the highest number of significant partners (overlap upregulation or downregulation q-values <10^–3^), with 26 and 25, respectively (Fig. 5a, Additional file 6: Table S8). Circuits involving them clearly dominate tumorigenesis in breast (16 and 12) and uterus carcinomas (12 and 5). One of the most significant partnerships of *MYC* in both breast and uterus cancer cells involves *ERBB2*, whose effect on the over-expression of the TF in HER2+ breast tumors [45, 46] has been noted before. The results shown here (Fig. 5b) suggest that this connection is also very prominent in tumors of the uterus. The circuit formed by *MYC* and the pair *CDKN2A*/*CDKN2B* also appears very significant in breast tumors (Fig. 5c), in coherence with the role that MYC amplification has on the enhancement of cell cycle de-regulation [47, 48]. Other very significant – and not so well known – partners of *MYC* in breast and uterus carcinomas include *NDRG1*, whose deficiency induces the epithelial-mesenchymal transition [49], *CARM1*, a methyltransferase known to methylate *NOTCH1* [50], *DDX5*, a key mediator in the inhibition of ribosomal biogenesis by *CDKN2A* [51], and *ZFP36L1*, a regulator of apoptosis [52]. Outside these two malignancies, *MYC* appears strongly connected to *STK11* in lung adenocarcinomas (Fig. 5d), a kinase related with global regulation of cell metabolism [53] among other processes. On the other hand, the list of *TP53* partners in four cancer types, as expected, is populated mainly by genes involved in the regulation of cell cycle and DNA damage detection and repair, such as *CCNE1*, *MDM2*, *MDM4*, *BRCA2*, *CHEK2*, *ATM*, and *ATR*.

Driver alterations of *KEAP1* in LUSC tumors result in the upregulation of the expression of a set of genes that very significantly (q-value = 1.42 × 10^–26^) overlap the upregulated targets of *NFE2L2* (Fig. 5e), corroborating that LoF mutations in the former cause tumorigenesis in lung squamous cells through the same pathway as gain-of-function mutations or amplifications of the latter. The disruption of this partnership involving the ubiquitination of *NFE2L2* through the *KEAP1*/*CUL3*/*RBX1* complex and its posterior proteasomal degradation, which leads to the constitutive activation of antioxidant response genes is known in several cancer types [54–56]. While the partnership in tumorigenesis of driver alterations of *KEAP1* and *NFE2L2* appears very prominent in LUSC tumors, *CUL3* seems a more outstanding partner in head and neck squamous carcinomas (Fig. 5a, Additional file 6: Table S8).

## Discussion

This work presents the first systematic characterization of the repertoire of mutational driver transcription factors in 28 human malignancies, as well as the collection of their targets most likely involved in tumorigenesis in 15 of these cancer types. Furthermore, exploiting the misregulation of the sets of significant targets of driver TFs, it highlights a set of connections with other driver genes, thus shedding light onto the mechanisms of tumorigenesis that underlie their alterations. Additional file 1: Figure S1 summarizes all the analyses carried out as part of this study.

There are caveats to both the detection of driver TFs and their targets involved in tumoriegensis and to the identification of driver circuits. The recognized incompleteness of the list of human TFs [11] and the catalog of driver genes – due to limitations built in the limit of detection of current methods [10, 13] – produces a repertoire of driver TFs that is not complete. Nevertheless, probably the predominant TFs involved in carcinogenesis upon somatic mutations in the tumor types studied are included within it. The shortcoming is more acute when it comes to the collection of target genes employed in the study. Although resulting from the combination of several databases exploiting different criteria to identify the targets of TFs, it is far from complete. (See further assessment of shortcomings in the detection of misregulated targets of driver TFs in Additional file 1: Tables S9 and S10.) In addition, note that the detection of differentially expressed targets of a TF is done previously to the detection of its partners and thus the set of samples bearing no driver mutations may actually include samples where a connected driver bears a mutation that result in the misregulation of the TF targets. As a result, the significance of the Mann–Whitney test comparing the expression of targets in both sets of samples may be diminished, causing the loss of some significant targets. False positive target genes, or targets not involved in tumorigenesis, may have also been included in the collection. Finally, the detection of drivers connected to TFs in the development of tumorigenesis is limited by the current extent of functional interaction networks.

Despite these limitations, the aforementioned outcomes of this work are relevant for the oncogenomics research community. Although previous works have identified cliques of drivers altered in a mutually exclusive manner across tumor samples [7, 8] – under the same rationale that this pattern signals convergent roads to tumorigenesis – no study to date has directly tested the hypothesis that alterations in connected drivers actually result in the misregulation of similar sets of targets. When the TFs and partners forming potential driver circuits probed in this study (with at least one significantly misregulated target gene shared by both the TF and its partner) are analyzed for mutual exclusivity of their pattern of alterations, only few of them reveal significant (See further details in Additional file 7: Table S11.) This is probably because the detection of potential downstream relationships between pairs of driver genes relying on the mutual exclusivity of their alterations is limited by the statistical power one can achieved from the frequency of somatic alterations. This limitation is probably less severe when such relationships are explored through the misregulation of downstream target genes. This highlights the interest of developing a bioinformatics method that exploits the overlap of misregulated target genes between pairs (or among groups) of drivers to reconstruct such relationships.

Besides the interest that the circuits of drivers detected in 15 different malignancies (Additional file 6: Table S8) may have for basic research dedicated to the emergence and evolution of cancer, they also have possible implications in the framework of pre-clinical research. It is reasonable to expect that tumors bearing alterations in driver genes connected in a circuit will follow similar evolution patterns, resulting in similar outcomes and exhibit similar response to a given therapeutic strategy. These circuits may thus represent a way of choosing therapies to indirectly target driver alterations in a tumor [10]. It is possible to envisage that this strategy to detect circuits of connected driver genes may be extended beyond this work focused on driver TFs, once more data on the functional consequences of LoF or activation of drivers – similar to the oncogenic signatures of MSigDB – become available.

In addition, the sets of significant targets of the driver TFs in the 15 tumor types studied here (Additional file 4: Table S6) constitute a source of data to formulate and test hypotheses on the tumorigenic process underlying them. As with the list of circuits, they have possible application to clinically-oriented research. The rationale in this case is that the accuracy of predictions on the outcome of tumors and their response to drugs could improve if the alteration status of significant targets is considered in addition to the status of driver TFs or their partners. With respect to the overlaps between the sets of significant targets of a TF in different tumor types, one can imagine the development of an ‘index of similarity’ between tumor types of different origin driven by alterations of the same gene, based on the overlap of their oncogenic signatures. This index could then be applied to predict the likelihood of success of repositioning a therapeutic strategy developed and tested on one tumor type [10] to another.

## Conclusions

The detection of overlapping sets of misregulated genes downstream pairs of drivers, or convergent misregulated targets allows to identify driver circuits, which is demonstrated as a proof of concept in this work. These driver circuits may provide clues to choosing therapies to indirectly target driver alterations in a tumor.

## Additional files

Additional file 1:
**PDF file containing description of all additional data as well as Figures S1-S4, and Tables S3, S4, S5, S9, and S10.** (PDF 1648 kb) Additional file 2: Table S1. List of families of driver TFs. (XLS 11 kb) Additional file 3: Table S2. Relative enrichment of driver TFs domains for somatic mutations across approximately 7,000 tumors. (XLS 79 kb) Additional file 4: Table S6. List of significant targets detected across 15 TCGA cohorts (XLS 466 kb) Additional file 5: Table S7. Overlap between sets of significant targets of a TF in pairs of tumor types. (XLS 69 kb) Additional file 6: Table S8. Driver circuits involving a TF and a partner driver tested across 15 tumor types. (XLS 49 kb) Additional file 7: Table S11. Mutual exclusivity of alterations of driver TF circuits. (XLS 24 kb)

## Abbreviations

ALL: Acute lymphoblastic leukemia
AML: LAML, Acute myeloid leukemia
BLCA: Bladder adenocarcinoma
BRCA: Breast adenocarcinoma
CLL: Chronic lymphocytic leukemia
COADREAD: Colorectal adenocarcinoma
CRF: chromatin regulatory factor
DLBCL: Difuse large B-cell lymphoma
ESCA: Esophageal carcinoma
GBM: Glioblastoma multiforme
HNSC: Head and neck squamous cell carcinoma
KIRC: Renal clear cell carcinoma
LGG: Low grade glioma
LHC: Liver hepatocellular carcinoma
LUAD: Lung adenocarcinoma
LUSC: Lung squamous cell carcinoma
MB: Medulloblastoma
MM: Multiple myeloma
NB: Neuroblastoma
NSCLC: Non-small cell lung cancer
OV: Ovarian cystadenocarcinoma
PAAD: Pancreatic adenocarcinoma
PRAD: Prostate adenocarcinoma
SCLC: Small cell lung cancer
SKCM: Skin cutaneous melanoma
STAD: Stomach adenocarcinoma
TF: Transcription factor
THCA: Thyroid adenocarcinoma
UCEC: Uterus endometrioid carcinoma
