# Supplementary material for: Circuits of cancer drivers revealed by convergent misregulation of transcription factor targets across tumor types
Source: Genome Med. 2016 Jan 20;8:6. doi: 10.1186/s13073-015-0260-1 (PMC4719577; doi:10.1186/s13073-015-0260-1)
Supplement: Additional file 1: — PDF file containing description of all additional data as well as Figures S1-S4, and Tables S3, S4, S5, S9, and S10. (PDF 1648 kb) [file 13073_2015_260_MOESM1_ESM.pdf]

Circuits of cancer drivers revealed by convergent miss-regulation of transcription factor targets across tumor types

Abel Gonzalez-Perez

Additional data

Table of Contents

Supplementary Figures....................2
Supplementary Tables....................14

Supplementary Figures

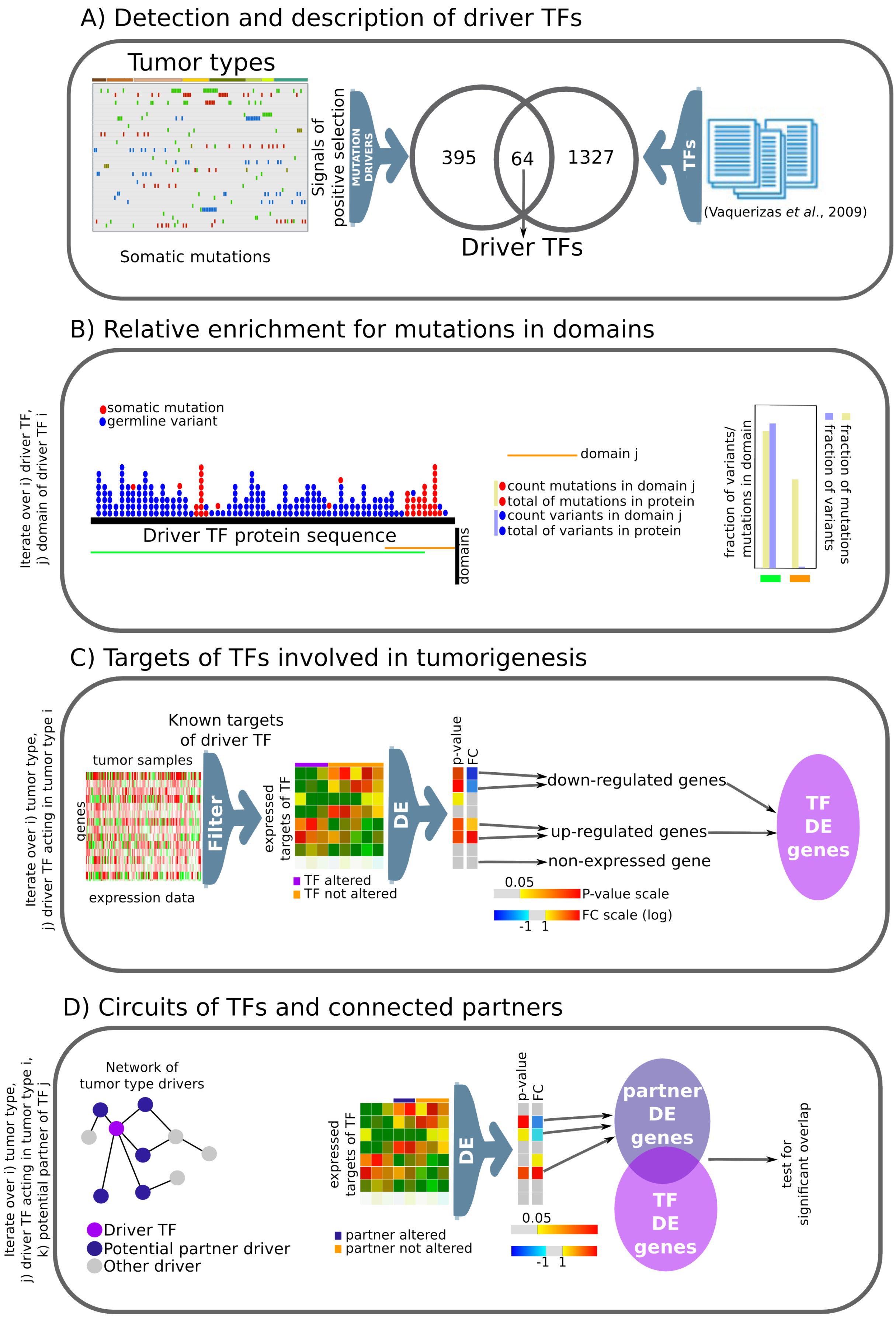
A) Detection and description of driver TFs
Tumor types
Somatic mutations
Signals of positive selection
MUTATION DRIVERS
395
64
1327
TFs
(Vaquerizas et al., 2009)
Driver TFs
B) Relative enrichment for mutations in domains
Iterate over i) driver TF, j) domain of driver TF i
somatic mutation
germline variant
Driver TF protein sequence
domains
domain j
count mutations in domain j
total of mutations in protein
count variants in domain j
total of variants in protein
fraction of variants/ mutations in domain
fraction of mutations
fraction of variants
C) Targets of TFs involved in tumorigenesis
Iterate over i) tumor type, j) driver TF acting in tumor type i
Known targets of driver TF
tumor samples
genes
expression data
Filter
expressed targets of TF
TF altered
TF not altered
DE
p-value
FC
down-regulated genes
up-regulated genes
non-expressed gene
0.05
P-value scale
FC scale (log)
-1 1
TF DE genes
D) Circuits of TFs and connected partners
Iterate over i) tumor type, j) driver TF acting in tumor type i, k) potential partner of TF j
Network of tumor type drivers
Driver TF
Potential partner driver
Other driver
expressed targets of TF
partner altered
partner not altered
DE
p-value
FC
0.05
-1 1
partner DE genes
TF DE genes
test for significant overlap

Supplementary Figure 1. Summary of all analyses carried out in this work

A) Detection and description of driver TFs. A list of genes driving tumorigenesis in different cancer types (drivers specific to each tumor type) identified through the combination of three signals of positive selection in their pattern of mutations in each cohort of tumors was obtained from reference 10. The intersection of these mutational drivers with an exhaustive list of human TFs produced a catalog of 64 driver TFs. (Note that only genes expressed in each tumor type can be nominated as drivers; therefore, all driver TFs are expressed in the tumor type where they act as drivers.)

B) Relative enrichment for mutations in domains. Lists of somatic mutations in tumors and germline variants in the human population affecting the 64 driver TFs were obtained from reference 10 and the ExAC database (see Methods). The latter were filtered by allelic frequency to keep only likely polymorphisms. Both sets were then mapped onto the protein coordinates of the driver TFs and the number of mutations and variants mapped to each domain in each driver TF were counted. The relative overrepresentation of mutations in each domain was finally computed via Fisher's exact tests.

C) Targets of TFs involved in tumorigenesis. Lists of known and predicted targets of 42 driver TFs were collected from several databases. The expression matrices of several TCGA cohorts of tumors (each representing one tumor type) were filtered using these lists, to retain only the expression of potential targets of each TF. The expression values of the targets of each driver TF across the tumor samples of a cancer type were probed for differential expression between the tumors where the TF is altered and the tumors where it is not altered. Targets with significant ($p<0.05$) Mann-Whitney test and log2 fold-change above 1 or below -1 were considered miss-regulated upon alterations of the TF (TF DE genes).

D) Circuits of TFs and connected partners. All (non-TF) drivers directly connected (through a functional interactions network) to each of the 42 driver TFs probed above were retrieved as potential circuit partners. The expression values of the targets of each driver TF across the tumor samples of a cancer type were probed for differential expression between the tumors where the potential partner is altered and the tumors where it is not altered, exactly as explained above for the TFs, which produced a set of partner DE genes. Finally, TF DE genes and partner DE genes were probed for significant overlap.

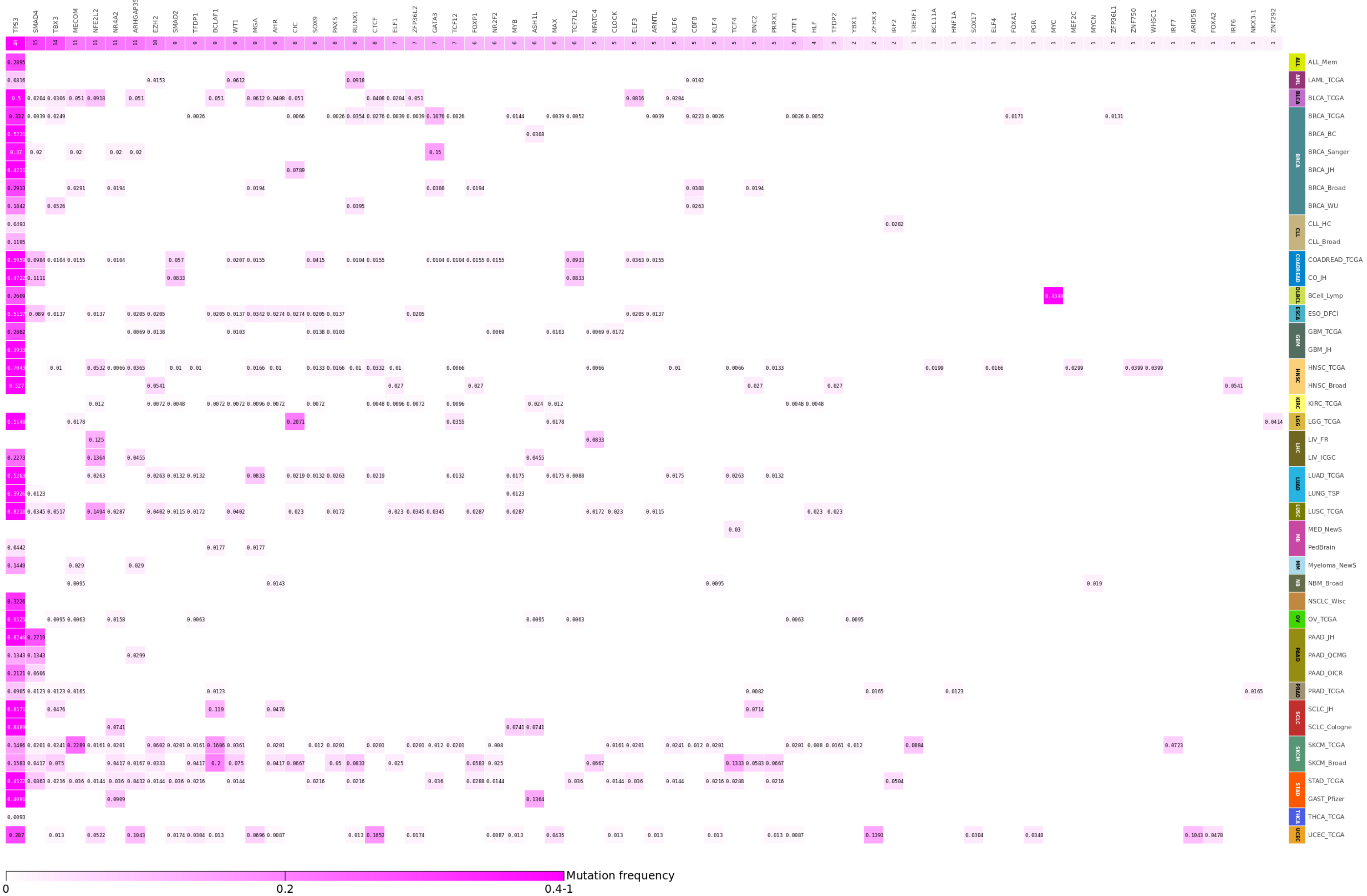

Supplementary Figure 2. Mutational frequency of driver TFs across 48 cohorts of tumors obtained from 28 cancer types. Rows and columns annotations are similar to Figure 1.

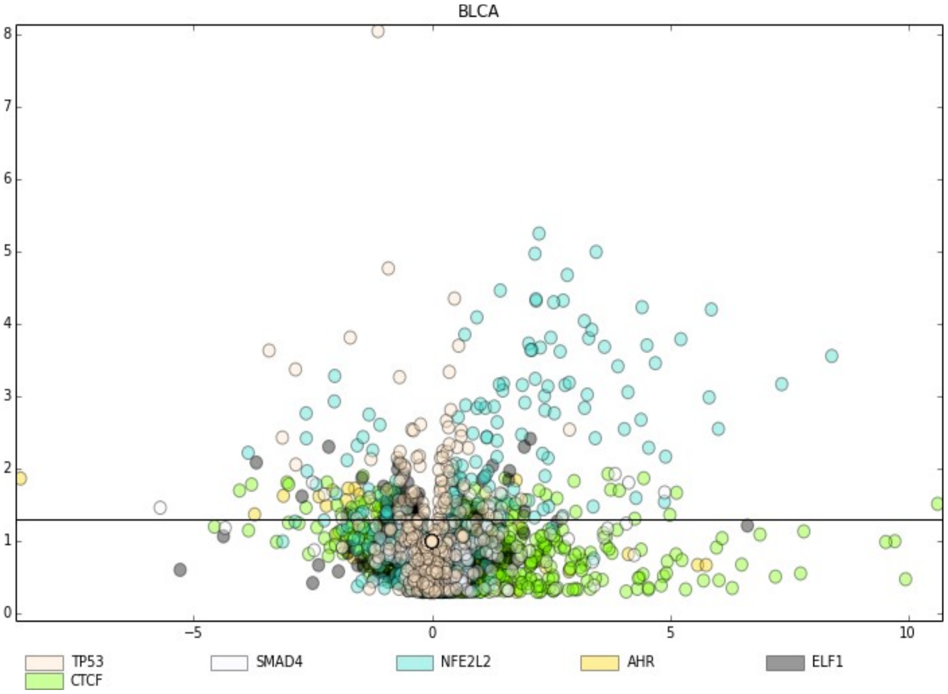
BLCA
8
7
6
5
4
3
2
1
0
−5
0
5
10
TP53
SMAD4
NFE2L2
AHR
ELF1
CTCF

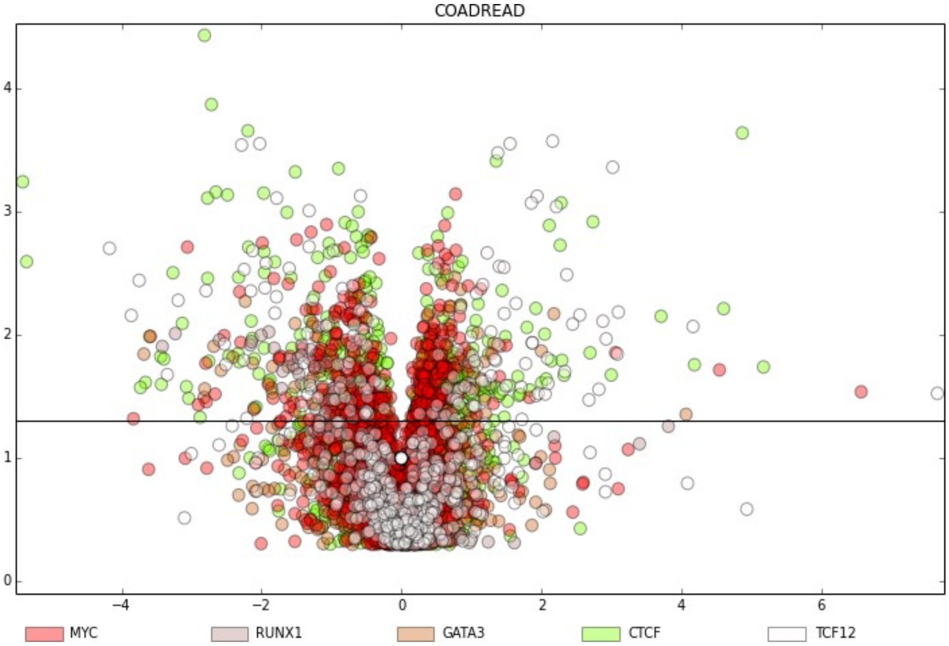
COADREAD
4
3
2
1
0
−4
−2
0
2
4
6
MYC
RUNX1
GATA3
CTCF
TCF12

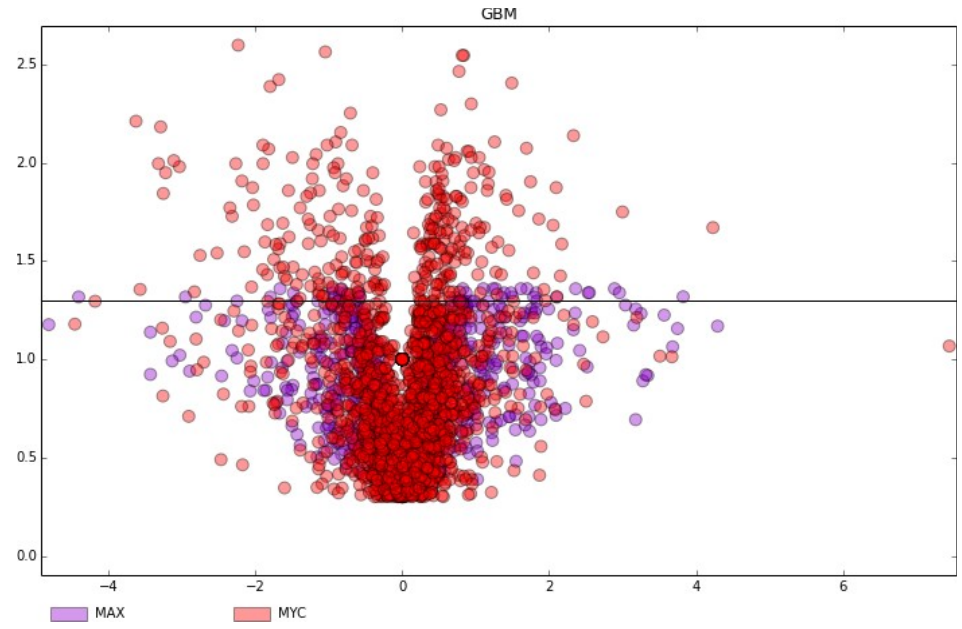
GBM
MAX
MYC

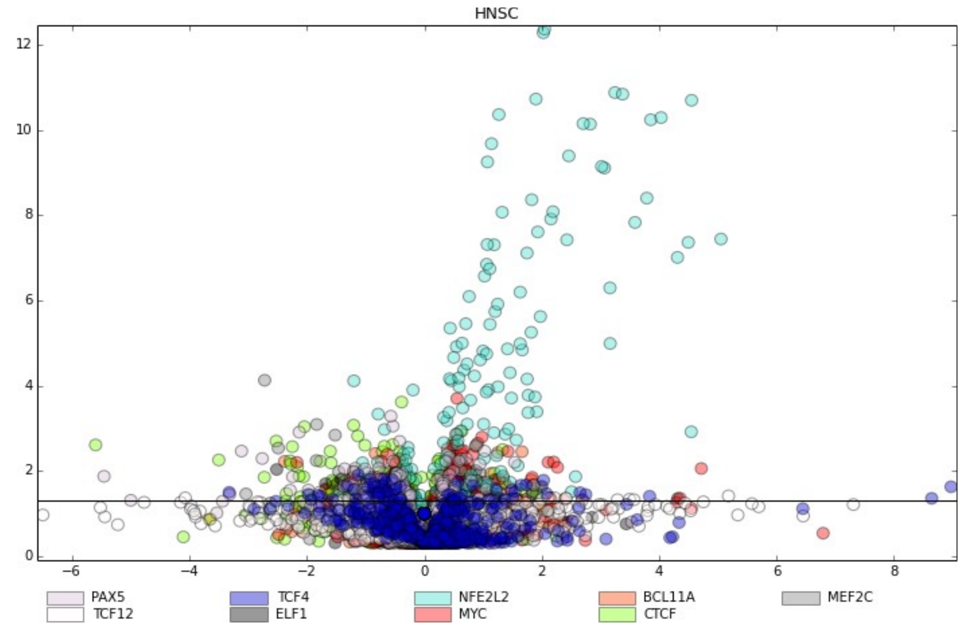
HNSC
PAX5
TCF12
TCF4
ELF1
NFE2L2
MYC
BCL11A
CTCF
MEF2C

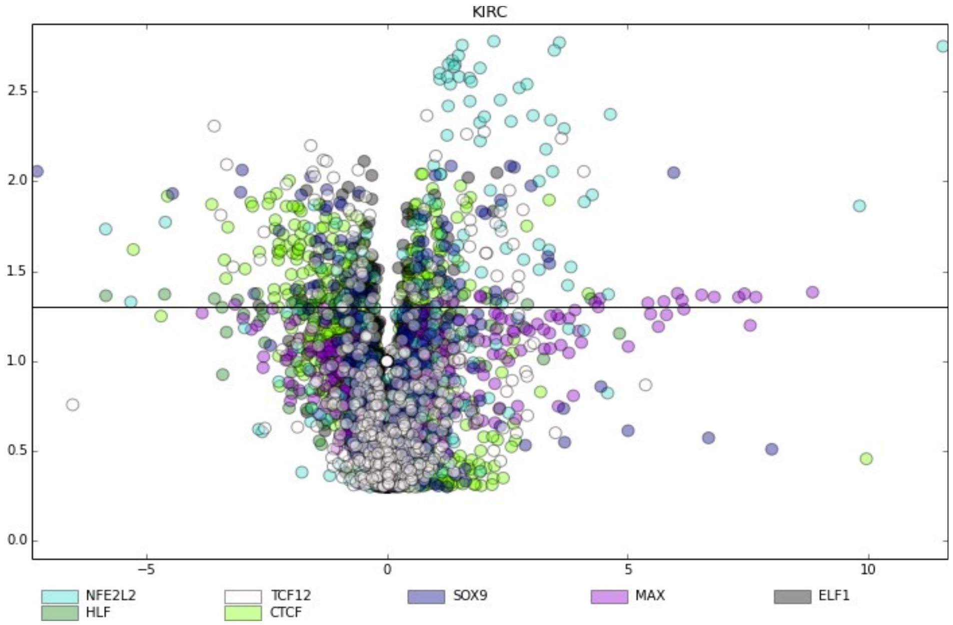
KIRC
2.5
2.0
1.5
1.0
0.5
0.0
−5
0
5
10
NFE2L2
TCF12
SOX9
MAX
ELF1
HLF
CTCF

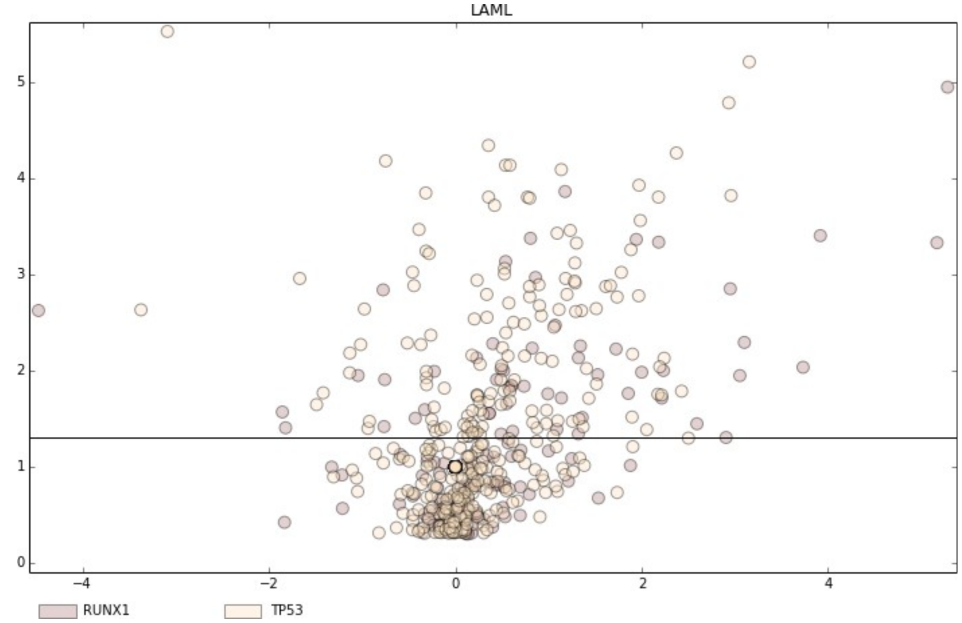
LAML
5
4
3
2
1
0
−4
−2
0
2
4
RUNX1
TP53

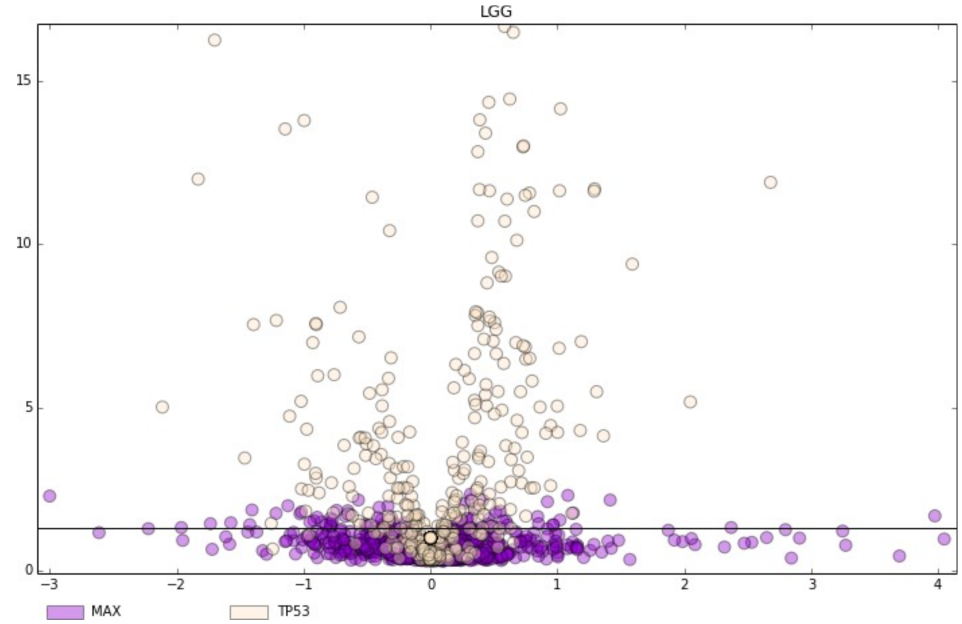
LGG
MAX
TP53

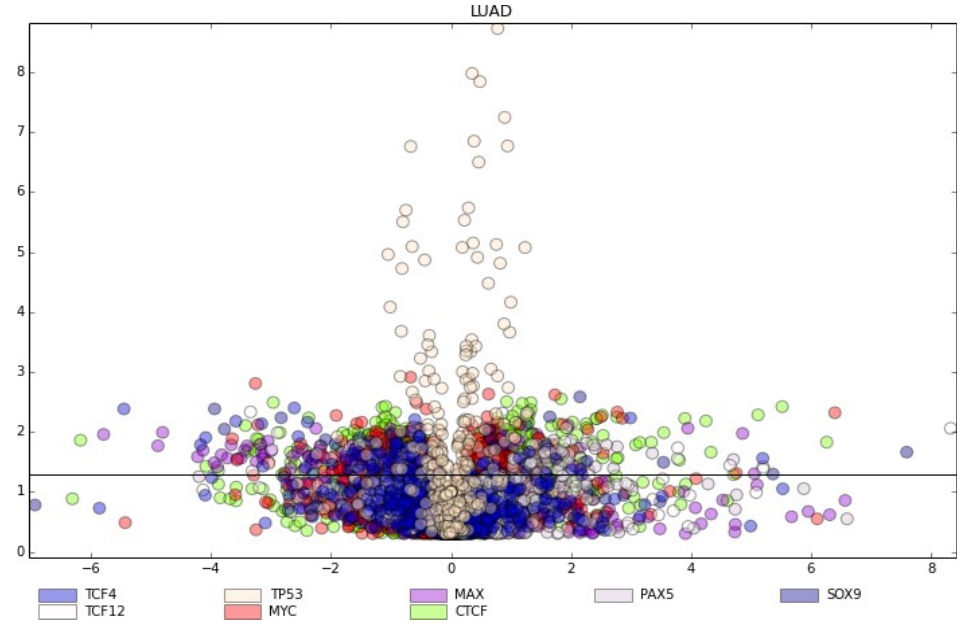
LUAD
TCF4
TCF12
TP53
MYC
MAX
CTCF
PAX5
SOX9

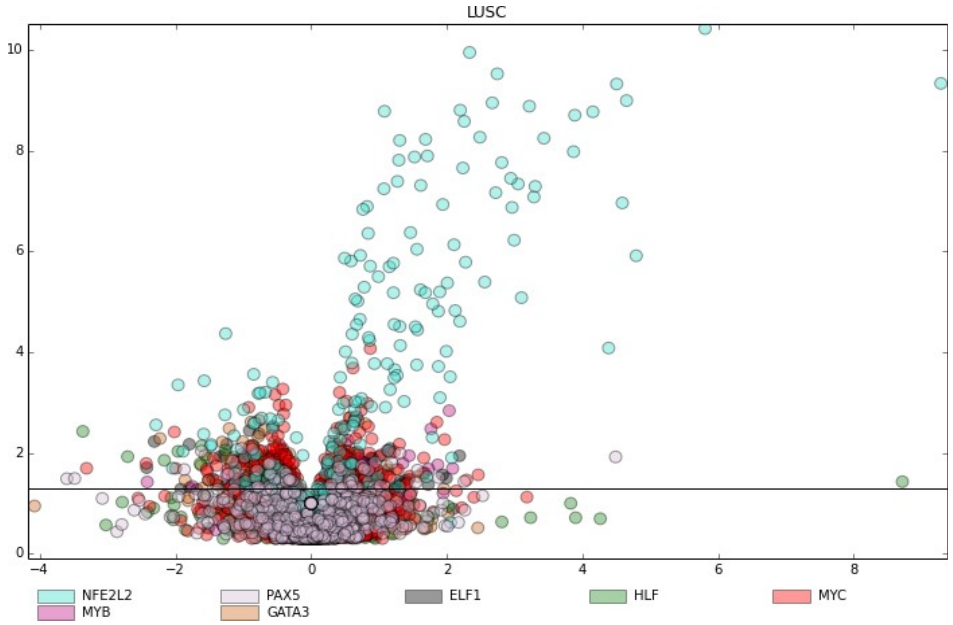
LUSC
NFE2L2
MYB
PAX5
GATA3
ELF1
HLF
MYC

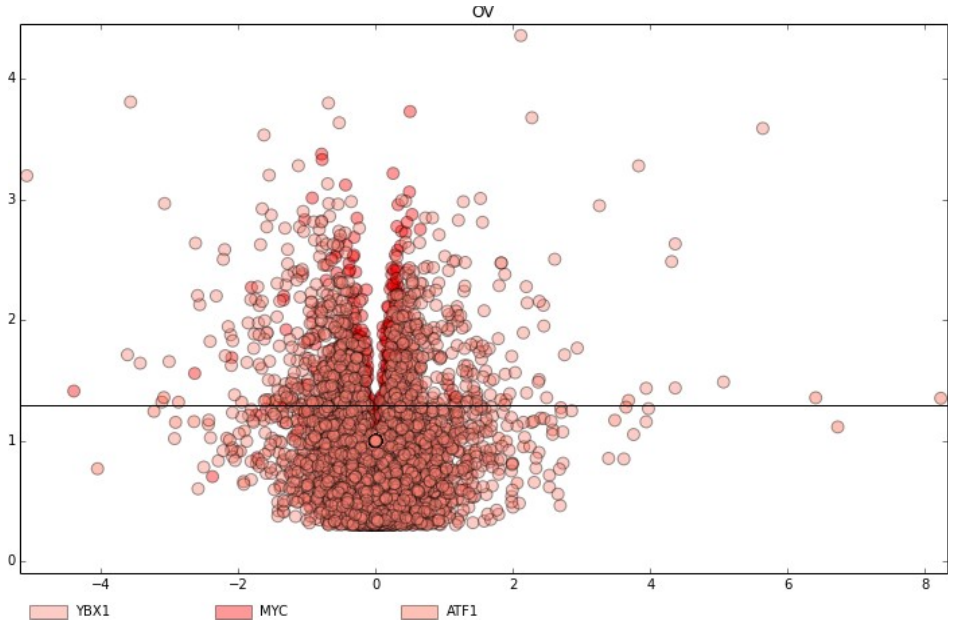
OV
YBX1
MYC
ATF1

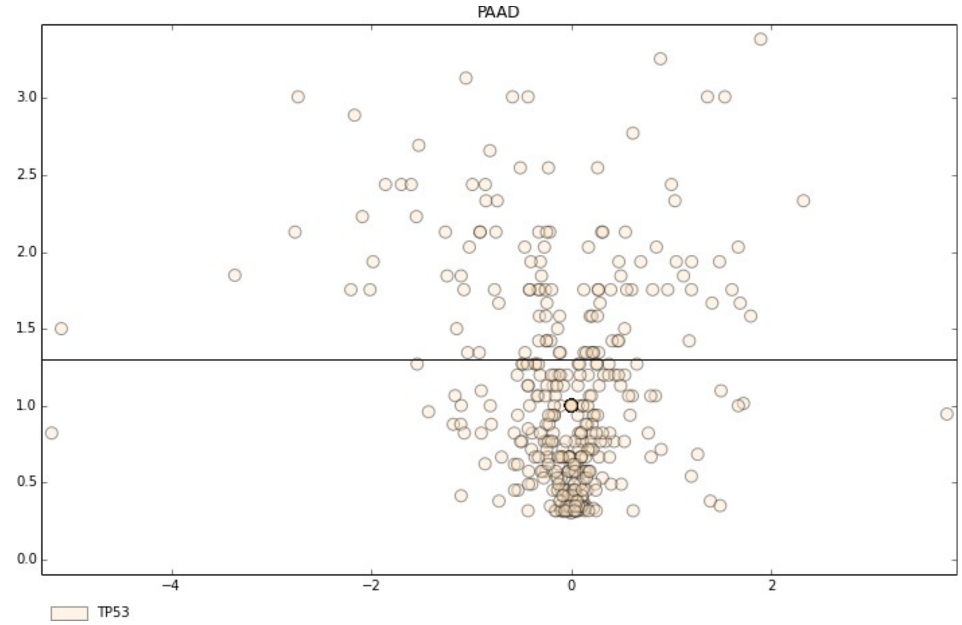
PAAD
3.0
2.5
2.0
1.5
1.0
0.5
0.0
−4
−2
0
2
TP53

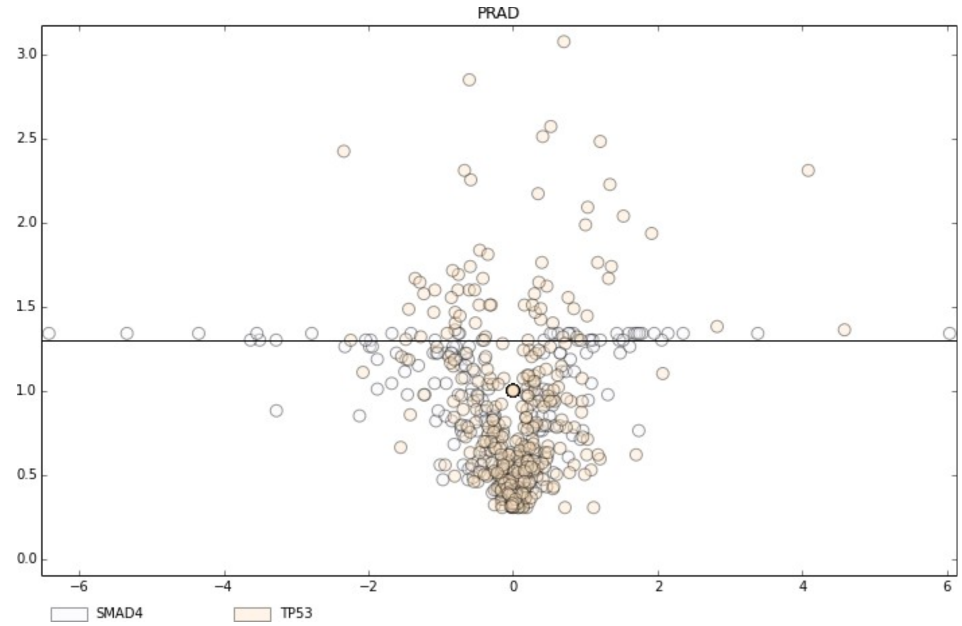
PRAD
3.0
2.5
2.0
1.5
1.0
0.5
0.0
−6
−4
−2
0
2
4
6
SMAD4
TP53

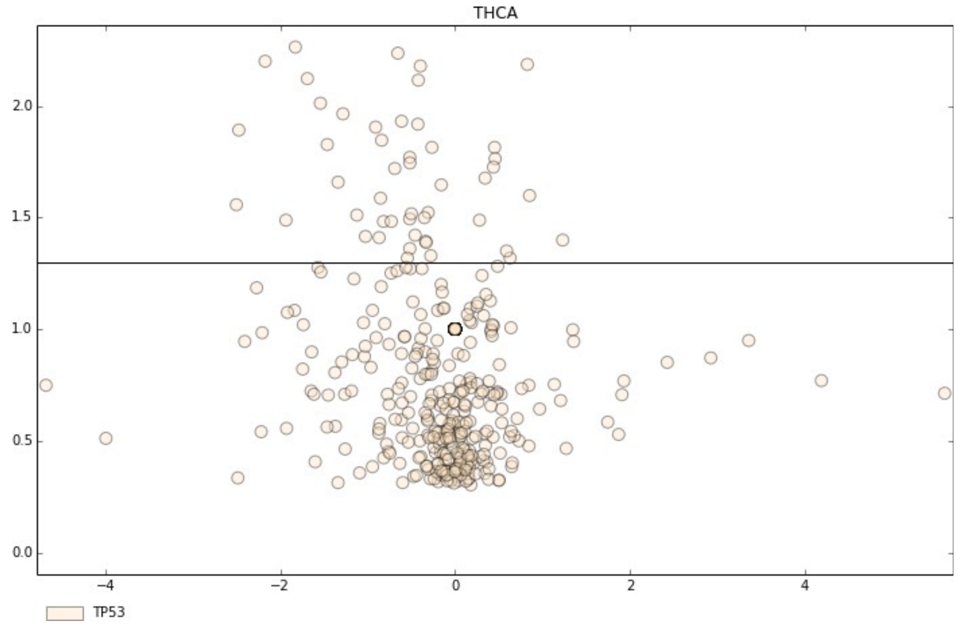

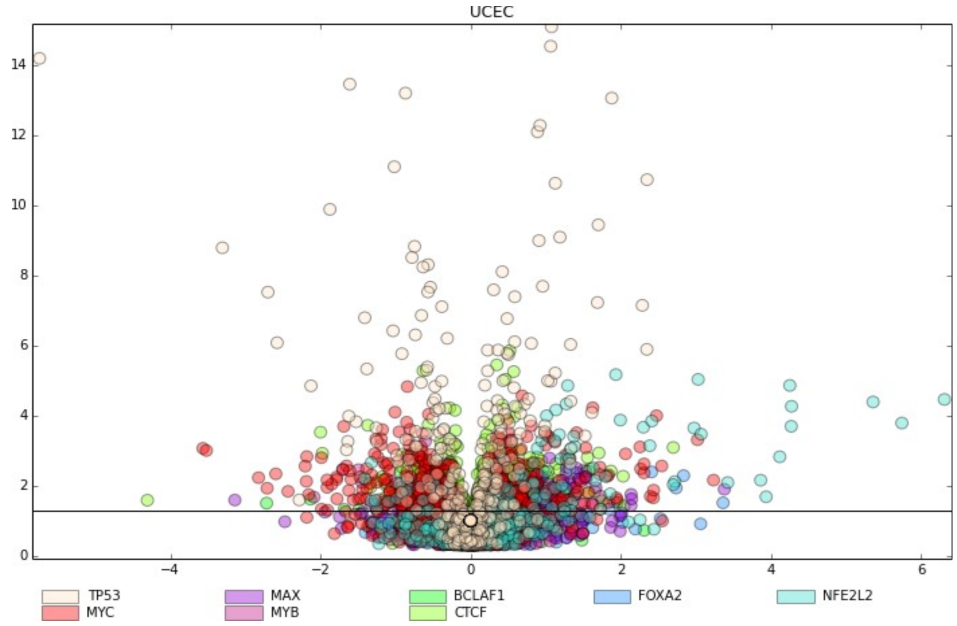

Supplementary Figure 3. Significant targets of driver TFs in 14 cohorts. Similar to Figure 3A.

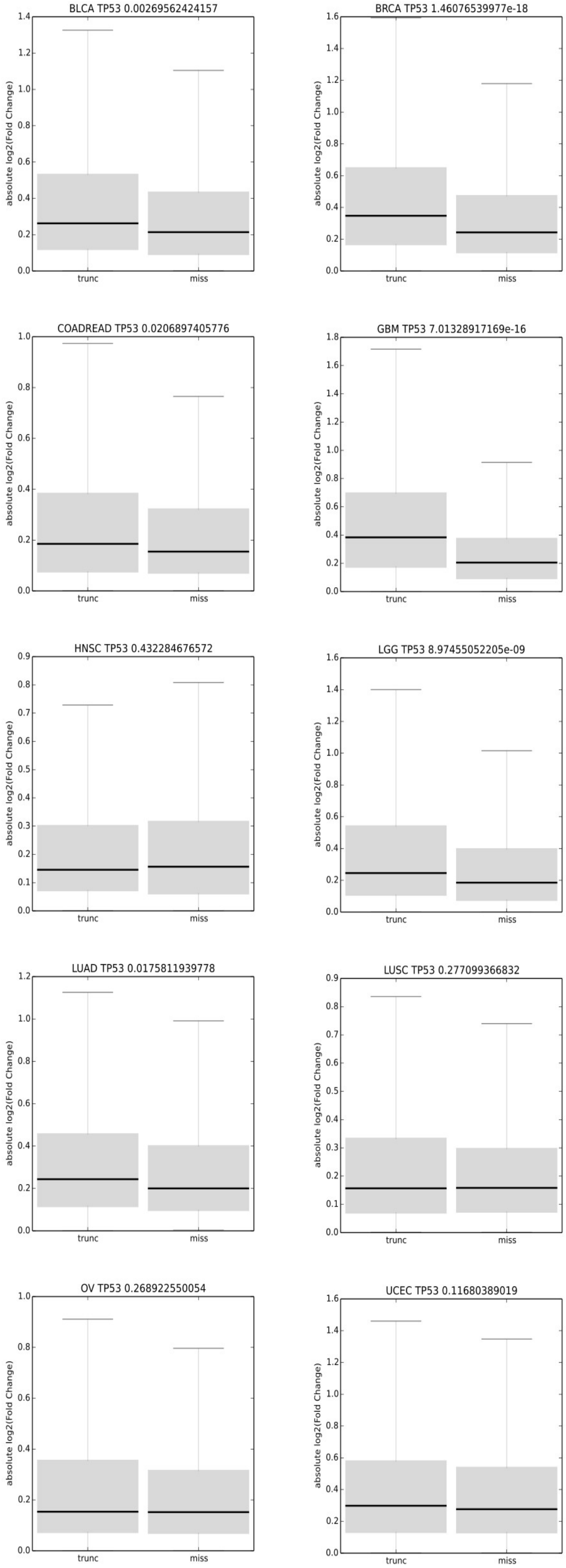

Supplementary Figure 4. Pooled comparison of the expression of TP53 targets in samples bearing TP53 truncating mutations and TP53 missense mutations in 10 tumor types.

Supplementary Tables

Supplementary Table 1 (Additional File 2). List of families of driver TFs

Supplementary Table 2 (Additional File 3). Relative enrichment of driver TFs domains for somatic mutations across ~7000 tumors.
Domain: domain name; TF: driver TF name; domain_length: length of domain; MDMr: fraction of mutations in domain with respect to the entire protein; VDVr: fraction of germline variants in domains with respect to the entire protein; -log(p-value): Fisher's -log(p-value)

Supplementary Table 3. Dataset of Transcription Factor targets collected from different sources

Source	PMID	URL	TFs	Targets	Interactions
HTRIdb	22900683	http://www.lbbc.ibb.unesp.br/htri/	283	18297	51869
pazar	18971253	http://www.pazar.info/	190	3227	5709
MSigDB	21546393	http://www.broadinstitute.org/gsea/msigdb/genesets.jsp?collection=TFT	283	12227	92542
ENCODE (Proximal/ Distal)	22955619	http://encodenets.gersteinlab.org/	110	9026	26070
			115	2167	19258

Supplementary Table 4. Number of targets collected for 42 driver TFs

TF	targets
YBX1	5963
MYC	2490
CTCF	2060
FOXA1	1720
GATA3	1619
MAX	1194
TCF4	797
PAX5	705
TCF12	682
ELF1	653
FOXA2	504
NFE2L2	493
BCL11A	469
TP53	419
SOX9	358
MYB	277
MEF2C	261
HLF	254
IRF7	252
SMAD4	244
ATF1	236
BCLAF1	228
AHR	221
RUNX1	169
IRF2	129
MYCN	63
HNF1A	39
PGR	12
WT1	8
NR2F2	5
KLF4	4
KLF6	3
TCF7L2	3
NR4A2	2
SMAD2	2
EZH2	2
FOXP1	2
WHSC1	1
IRF6	1
TRERF1	1
NKX3-1	1
ZFHX3	1

Supplementary Table 5. TCGA datasets of genomic alterations across tumor types

Dataset name	Tumor type	Mutations samples	CNA samples	Expression samples
BLCA	Bladder carcinoma	99	125	97
BRCA	Breast carcinoma	771	874	818
COADREAD	Colorectal adenocarcinoma	224	575	264
GBM	Glioblastoma multiforme	291	563	162
HNSC	Head and neck squamous cell carcinoma	306	306	304
KIRC	Renal clear cell carcinoma	417	452	429
LAML	Acute myeloid leukemia	196	197	174
LGG	Lower grade glioma	170	181	206
LUAD	Lung adenocarcinoma	230	356	354
LUSC	Lung squamous cell carcinoma	178	342	221
OV	Ovary cystadenocarcinoma	316	566	264
PAAD	Pancreas adenocarcinoma	34	48	42
PRAD	Prostate adenocarcinoma	83	172	143
THCA	Thyroid carcinoma	323	352	427
UCEC	Uterus endometriod carcinoma	248	493	334
**Total**		**3886**	**5602**	**4239**

Supplementary Table 6 (Additional File 4). List of significant targets detected across 15 TCGA cohorts
Tumor type: tumor type acronym; TF: driver TF name; target: target gene name; -log10(p-value): Mann-Whitney -log(p-value); log2(FC): log2(fold-change)

Supplementary Table 7 (Additional File 5). Overlap between sets of significant targets of a TF in pairs of tumor types
TF: driver TF name; j: Jaccard's index; p: Fisher's p-value; q: Fisher's corrected p-value; ttype1: tumor type 1 acronym; ttype2: tumor type 2 acronym

Supplementary Table 8 (Additional File 6). Driver circuits involving a TF and a partner driver tested across 15 tumor types
TF: driver TF name; partner: driver partner name; ptcga_dn: Fisher's pvalue of overlap between targets down-regulated resulting from mutations in TF of partner; ptcga_up: Fisher's pvalue of overlap between targets up-regulated resulting from mutations in TF of partner; qvals_up: corrected p-value for overlap of up-regulated targets; qvals_dn: corrected p-value for overlap of down-regulated targets; ttype: tumor type acronym

Supplementary Table 9. Overlap between the list of targets extracted from databases for each driver TF and the set of genes misregulated in cancer cells bearing a knock-down of the same driver TF.

TF	Targets (mapped to LINCS genes)	LINCS misregulated targets	Odds-ratio	P-value
HNF1A	36	3	539.13	0
IRF2	129	27	175.32	0
RUNX1	169	39	137.21	0
AHR	219	44	102.72	0
BCLAF1	228	35	92.52	0
ATF1	235	56	100.44	0
BRCA1	238	99	129.43	0
SMAD4	244	79	108.60	0
IRF7	252	42	85.09	0
HLF	254	89	108.93	0
MEF2C	261	74	95.69	0
MYB	276	97	100.37	0
TP53	417	147	67.19	0
NFE2L2	485	130	50.65	0
FOXA2	499	83	43.19	0
ELF1	648	102	32.97	0
TCF12	671	122	32.72	0
PAX5	694	40	27.24	0
TCF4	767	166	30.07	0
MAX	1132	410	25.67	0
GATA3	1575	574	19.19	0
FOXA1	1593	337	15.11	0
CTCF	2008	529	12.48	0
MYC	2209	1155	19.06	0
YBX1	5961	1021	3.79	0

To compute these overlaps I first downloaded from the LINCS project (**http://api.lincscloud.org/a2**) the lists of genes misregulated (100 up-regulated and 100 down-regulated) in cancer cells in which the mRNA of each driver TF had been knocked down, with respect to control cells. I then merged the lists of misregulated genes in all cancer cells obtained from LINCS. Next, I mapped the targets of each TF to all genes probed by misregulation in LINCS. Finally I computed the significance of the overlap between these sets through a Fisher's exact test. The table only shows the results for TFs with at least 20 targets mapped to LINCS genes.

Supplementary Table 10. Specificity of the differential expression analysis.

TF	ttype	Z_expected_DE_genes	p_DE_targets	Class
TP53	LGG	83	0.0824	known
TP53	BRCA	62	0.6816	putative_unknown
NFE2L2	LUSC	38.1164541589	0	known
KLF6	LUAD	31.6666666667	0.0073	known
TP53	UCEC	30.4255531707	0.3157	putative_unknown
NFE2L2	HNSC	23.7049638512	0	known
NFE2L2	BLCA	18.0175233464	0	known
NFE2L2	UCEC	12.8648970538	0	known
TP53	PAAD	12.6889735698	0.0068	known
RUNX1	LAML	12.3173515508	0	known
RUNX1	COADREAD	9.7192739295	0.9994	putative_unknown
TP53	LUAD	9.0990715709	0.4052	putative_unknown
TP53	LAML	8.5440936381	0.0452	known
HLF	BRCA	8.2158383627	0.0057	known
NFE2L2	KIRC	6.9412967777	0	known
MYB	BRCA	6.5	0.7958	putative_unknown
TP53	PRAD	5.7695803006	0.1444	putative_unknown
HLF	KIRC	5.1547009721	0.835	putative_unknown
TP53	BLCA	4.8962976112	0.0795	putative_unknown
TP53	HNSC	4.3548148998	0.2039	putative_unknown
MEF2C	HNSC	4.2	0.5265	putative_unknown
TP53	OV	3.9196474793	0.1998	putative_unknown
WT1	LAML	3.709704134	0.0044	known
ATF1	OV	3.6015645651	0.9018	putative_unknown
BCLAF1	UCEC	3.4325139812	0.4335	putative_unknown
NR4A2	OV	3	0.003	known
KLF6	BLCA	3	0.0045	known
TCF7L2	LUAD	3	0.0091	known
KLF4	UCEC	3	0.0192	known
TP53	GBM	2.9880715233	0.0974	putative_unknown
SOX9	KIRC	2.9824794097	0.4037	putative_unknown
SMAD4	PRAD	2.831042407	0.0346	known
SMAD4	PAAD	2.6616331806	0.2346	putative_unknown
SMAD4	COADREAD	2.5533076283	0.1227	putative_unknown
MYB	UCEC	2.523375565	0.0041	known
SOX9	COADREAD	2.0647416049	0.5838	putative_unknown
MYB	LUSC	1.9330913339	0.0103	known
TP53	LUSC	1.6299670689	0.2225	possibly_unspecific
TP53	THCA	1.014999207	0.6398	possibly_unspecific
TP53	COADREAD	1	0.9277	possibly_unspecific
BCL11A	HNSC	0.8389938108	0.0089	possibly_unspecific
SOX9	LUAD	0.8145332746	0.9323	possibly_unspecific
RUNX1	HNSC	0.6546536707	0.278	possibly_unspecific
RUNX1	UCEC	0.6546536707	0.3673	possibly_unspecific
AHR	UCEC	0.4986168715	0.664	possibly_unspecific
HLF	LUSC	0.4506059091	0.5242	possibly_unspecific
SMAD4	LUSC	0.3821578532	0.5552	possibly_unspecific
AHR	BLCA	0.2790059343	0.6557	possibly_unspecific
AHR	HNSC	0.2526455763	0.5263	possibly_unspecific
BCLAF1	KIRC	0.1297821967	0.9333	possibly_unspecific
SMAD4	BRCA	0.0211809271	0.29	possibly_unspecific
ATF1	BRCA	-0.1662963908	0.3441	possibly_unspecific
SMAD4	BLCA	-0.2543739546	0.1867	possibly_unspecific
RUNX1	BRCA	-0.2904089348	0.5226	possibly_unspecific
ATF1	KIRC	-0.3323176901	0.3376	possibly_unspecific
MYB	LUAD	-0.8180438565	0.1054	possibly_unspecific
SOX9	HNSC	-0.8849631314	0.4227	possibly_unspecific
BCLAF1	BLCA	-0.9857962275	0.872	possibly_unspecific
ATF1	UCEC	-1.6606633454	0.8464	possibly_unspecific

To produce this table, I first randomly sampled groups of genes of the same size as the starting number of targets annotated for each TF. Then, I checked how many of these genes appeared differentially expressed between the samples with alterations of the TF and the samples where the TF is unaltered. I iterated this process 10000 times and computed an *ad hoc* p-value (**p_DE_targets**) of the representativity of the TF targets as the amount of these iterations where the number of recorded differentially expressed targets of the TF was larger than the number of differentially expressed genes. (I limit the analysis to TFs with less than 500 targets, to assure enough difference in sampling the groups of random genes.)

Low p-values, thus denote TFs for which the differential expression analysis detects mostly genes within their lists of collected targets. On the other hand, TFs-tumor types combinations with p-values close to 1 represent cases in which differentially expressed genes are distributed both within and outside the collected targets. This may be due to i) incompleteness of the collections of targets of these TFs –mainly indirect targets–, ii) dramatic changes in gene regulation that take place in tumorigenesis or iii) spurious results from the differential expression analysis. To distinguish between these two possibilities I then carried out a second analysis to estimate the expected number (as fraction of the number of known targets of the TF) of differentially expressed genes to be detected given the number of samples where the TF bears driver alterations in the tumor type under analysis. Briefly, for each TF-tumor type combination, I randomly assigned the samples 100 times to two groups, one of them composed of the same number as the samples with driver alterations of the TF. I then probed the differential expression of a random set of genes of the same size as the known targets of the TF. Finally, by integrating the counts of differentially expressed genes across these 100 iterations, and comparing them to the observed number of differentially expressed targets of the TF, I computed a Zscore (**Z_expected_DE_genes**). This Zscore thus measures the significance of the number of observed differentially expressed targets given the expected number of differentially expressed genes from factors in principle not associated to alterations in the TF –i.e., such as massive changes in transcriptional program due to tumorigenesis.

According to the combination of the p_DE_targets and the Z_expected_DE_genes I classified TF-tumor type combinations into three groups (column **Class**). Those in the 'known' group possess both a significant p_DE_targets ($p<0.05$) and a significant Z_expected_DE_genes ($Z>1.96$) and therefore correspond to cases where the fraction of differentially expressed targets are significantly higher than expected from factors not necessarily associated to TF alterations and also significantly higher than the number of differentially expressed genes outside the list of TF targets. 'putative_unknown' targets of TFs have a significant Zscore, but non significant p, pointing probably to an important number of yet undiscovered targets which become misregulated upon alteration of the TF. Finally, the set of 'possibly_unspecific' targets of TFs correspond to cases where the fraction of differentially expressed targets is neither significantly higher than expected from groups of random genes nor greater than expected from factors not associated to alterations in the TF. Differential expression detected within the targets of these TFs cannot therefore be linked exclusively to the alteration of the TF.

Supplementary Table 11 (Additional File 7). Assessment of the mutual exclusivity of alterations of driver TF circuits

Two methods (mutex and Comet; see Methods) that compute the mutual exclusivity of alterations were used on all TF driver circuits explored in this study with at least one target gene in common between the TF and its partner. The overlap between the fraction of these circuits that exhibit a significant overlap of targets (signif_circ in the Table) and those detected as pairs with significant mutually exclusive alterations (signif_mutex, signif_both, signif_comet) is rather small (Fisher's p-value=0.22). This is because the overlap of significantly miss-regulated targets and the mutual exclusivity of alterations are orthogonal ways of assessing the relationships between driver genes. While the former relies on the information of targets, and their expression in the same samples where the mutational and CNA status of the driver TFs and partners is assessed, and cannot be used if this is not available, the latter only requires the knowledge of these mutational and CNA status of the drivers. On the other hand, the overlap of the miss-regulation of targets theoretically could detect convergent alterations between driver TFs and their partners that fall below the threshold of significance of mutual exclusivity (as suggested by the results of the Table). Thus, a bioinformatics method developed using the rationale presented in this study may represent a good alternative to mutual exclusivity to detect such relationships between driver genes.
